# Supplementary material for: Mineralocorticoid Receptor Signaling in Peripheral Blood Cells in Patients with Multiple Sclerosis
Source: Int J Mol Sci. 2024 Aug 15;25(16):8883. doi: 10.3390/ijms25168883 (PMC11354852; doi:10.3390/ijms25168883)
Supplement: Supplementary file 1 [file ijms-25-08883-s001.zip › ijms-3108421-supplementary.pdf]

Supplementary Material

S1 Supplementary to Subjects, Materials and Methods

S1.1. Gene expression analysis (microarray)

To increase validity at a low number of probands (N=3), we chose a homogeneous cohort of three healthy young men of approximately the same age and with similar lifestyle (mean age 27±1, non-smokers, Caucasian background). Cell culture experiment was run as outlined in the main text and cells of each subject were cultured in four different conditions (administration of aldosterone, cortisol, aldosterone and cortisol or vehicle, respectively) to generate twelve samples. Two Illumina HT12-Beadchip with twelve arrays each were consecutively used to investigate gene expression levels, one serving as a technical replicate to the other. The total amount of RNA hybridized was 651 ± 97.2 ng per array for subject one, 1215 ± 71.4 ng per array for subject two and 748 ± 125.5 ng per array for subject 3. Hybridization to the microarray as well as standardization of data using Genome Studio (Version 2011.1) was performed at the Core Unit DNA Technologies (Core Facilities of the Medical Faculty, University Leipzig). Data was provided in a set of non-normalized and quantile-normalized data with and without subtracted background respectively as well as original IDAT-data and excel files. The discussed data (non-normalized and quantile-normalized data with subtracted background) are available on NCBI's Gene Expression Omnibus [28] through GEO Series accession number GSE162695 (https://www.ncbi.nlm.nih.gov/geo/query/acc.cgi?acc=GSE162695).

S1.2 Identification of MR target genes

With the intention of identifying MR marker genes we analyzed the abundance of transcripts using two Illumina HT12-Beadchip microarray. Gene lists were created using Microsoft Access. We hereby focused on induced gene expression. In short, our strategy was designed to find consistent up-regulation rather than detecting the highest fold-induction. We decided to process each healthy subjects' data set individually and compare the resulting gene lists for consistency. Also, we ruled out a strong regulation by cortisol and therefore avoided a highly induced expression in one subject to override smaller but more consistent effects in other genes.

We primarily processed the quantile, non-background-subtracted data. For better understanding of the following steps please see the explanatory illustration in Figure S3. In a first step vehicle signals were subtracted from aldosterone signals for each subject individually. To find targets influenced more strongly by aldosterone than by cortisol, cortisol signals were also normalized to vehicle and subtracted from the normalized aldosterone signals. Negative values for the normalized data sets (e.g. suppression by administration of aldosterone or cortisol) were excluded. To avoid an influence of genes downregulated by cortisol, all those genes with strong negative values (<-50) for "cortisol minus vehicle" were excluded. To further select strongly induced genes upon aldosterone stimulation we set the threshold for „aldosterone minus vehicle" to 40; signals normalized to vehicle as well as p-detection values for cortisol and aldosterone probe sets are shown in the appendix (Table S2, Table S3, Table S4). As an internal control, several computational variations were compared. First, the same data (quantile non-background-subtracted data) was logarithmized and subsequently subtracted following the strategy outlined above. Second, the quantile background-subtracted data underwent the initial analysis without logarithmization. These gene lists generated in the second approach yielded identical result lists. In a last control, instead of subtracting, we calculated ratios using the quantile background-subtracted data. Here, a minimal discrepancy occurred in comparison of subtraction versus ratio computation of the quantile background subtracted data sets (inconsistent in 21 genes). Last, we confirmed that upregulation of genes was at least qualitatively consistent among the three subjects

(see Table S5). Those genes, which were upregulated in all three gene lists underwent further investigation.

A total of 13 genes were selected for subsequent validation using PBMCs from separate samples subject to the same incubation procedure as described above, followed by qRT-PCR with dedicated, custom-designed primers (Figure 2). Closed testing procedure [52] after six samples was performed to correct for multiple testing and was positive for OTUD1, Serpin B2, CD83, IL1 B. Further analysis with Student's t-test showed a significant change only for OTUD1.

S1.3. Primer sequences for qPCR

Table S1. Primer sequences of custom designed primers as used for qRT-PCR validation of putative MR target genes. Left column shows gene names, right column the respective oligonucleotide sequences.

	**SEQUENCES**
**CCL3**	Forward: 5′-gcttcgcttggttaggaaga-3′<br>Reverse: 5′-tgctgcttcagctacacctc-3′
**CD83**	Forward: 5′-acagagcggagattgtcctg-3′<br>Reverse: 5′-gagaaaagctcgttccatgc-3′
**CYP1B1**	Forward: 5′-attctgcctgcactcgagtc-3′<br>Reverse: 5′-aacgtaccggccactatcac-3′
**GAPDH**	Forward: 5′-gagtcaacggatttggtcgt-3′<br>Reverse: 5′-gaggtcaatgaaggggtcat-3′
**IER3**	Forward: 5′- cgccgaagtctcacacagta-3′<br>Reverse: 5′-aggagaagaaatggggagga-3′
**IL-1B**	Forward: 5′-gacaaatcgcttttccatcttc-3′<br>Reverse: 5′-gggcctcaaggaaaagaatc -3′
**IL-8**	Forward: 5′-aaggaaaactgggtgcagag-3′<br>Reverse: 5′- gcttgaagtttcactggcatc-3′
**KCTD6**	Forward: 5′-ttgaagacgcatcactggag-3′<br>Reverse: 5′-cgtcaatgtggtgagagacg-3′
**NR3C2 (MR)**	Forward: 5′-cccataatggcatcctgaag-3′<br>Reverse: 5′-cattccagaaaatgtatcaagctc-3′
**OSM**	Forward: 5′-ccctgcagtgctctctcagt-3′<br>Reverse: 5′-ataggcagctgctcgaaaga-3′
**OTUD1**	Forward: 5′-acgacaactggtgcaaacaa-3′<br>Reverse: 5′-cccagggtgtaaggttttga-3′
**PTGS2**	Forward: 5′-ggtcaatggaagcctgtgat-3′<br>Reverse: 5′-gctggaacatggaattaccc-3′
**SERPIN B2**	Forward: 5′-cagatgaaattgccgatgtg-3′<br>Reverse: 5′-gccattttgtctttgctggt-3′
**SLC16A6**	Forward: 5′-cagtgctccgaagatgacaa-3′<br>Reverse: 5′-agtcactgcagttgcttcca-3′
**SLC25A24**	Forward: 5′-ccatgactccagggtttacag-3′<br>Reverse: 5′-atgaaggcttgggagcttttt-3′

S2 Supplementary Figures

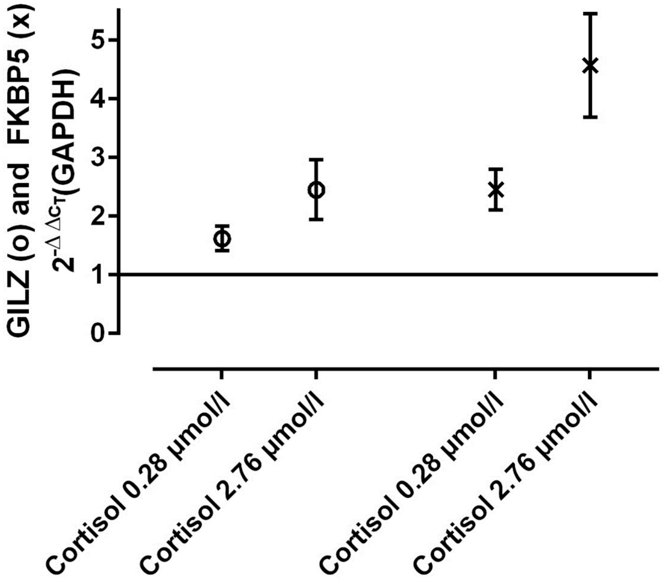

Figure S1. Dose-finding experiment for cortisol. Cell culture was performed with PBMC of three healthy male subjects (N=3). Gene expression levels of GR downstream-targets GILZ (indicated by "o" in the left section of graphs) and FKBP5 (indicated by "x" in the right section of graphs) upon stimulation with cortisol. Dosage as indicated on x-axis. Data is shown as mean and standard error of the mean (SEM).

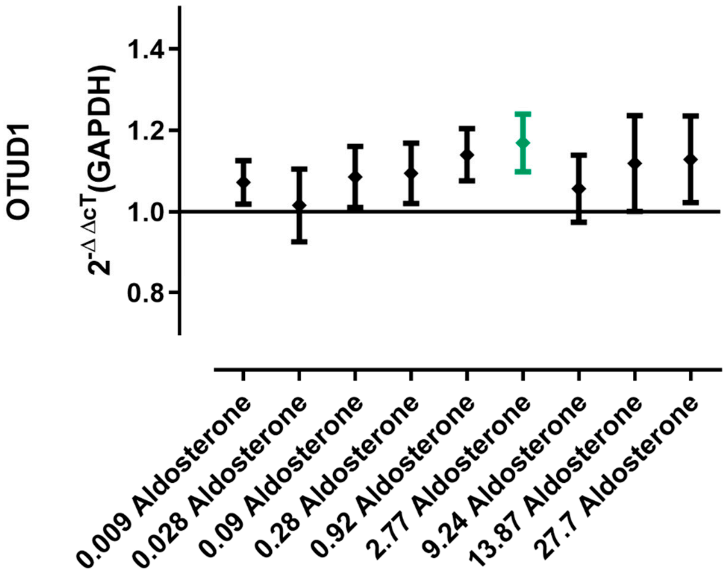

Figure S2. Dosefinding experiment for aldosterone. Cell culture was performed with PBMC of nine healthy subjects (N=9). Gene expression levels of MR marker gene OTUD1 upon stimulation with aldosterone. Dosage as indicated on x-axis in nmol/l. Data is shown as mean and standard error of the mean (SEM).

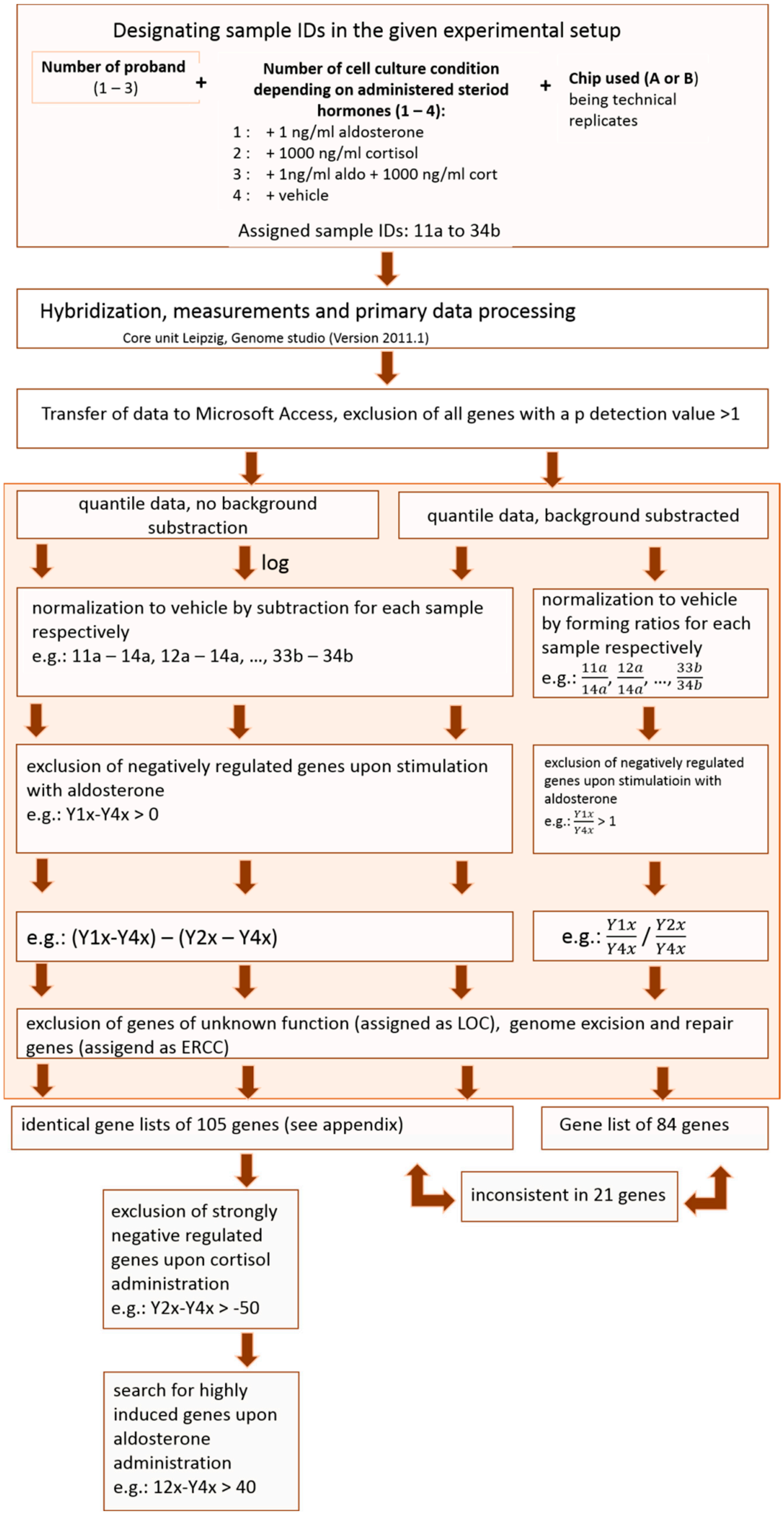

Figure S3. Workflow for the first strategy of microarray analysis. The light orange box marks the main calculation steps taken. The four columns of brown arrows indicate respective data processing of the two primary data sets. At last the four independent calculations led to comparable/identical gene lists.

Supplementary Table

Table S2. Resulting gene list for proband 1 following the strategy as outlined in paragraph S3.1 and depicted in Figure S3. For understanding of sample IDs in column titles, refer to Figure S3. In short: ‚a' or ‚b' indicates MicroArray used (b being a technical replicate of a), the first digit designates the number of the proband and the second digit the incubation condition (1 = aldosterone, 2 = cortisol, 4 = vehicle). Normalisation to vehicle is indicated by adding the vehicles abbreviation respectively, e.g. Probe_a11_14. Further processing of data sets normalised to vehicle is designated per MicroArray and proband, e.g. aAld-aCort 1 meaning cortisol signals were subtracted from aldosterone signals. P detection values of <0.001 are rounded down to zero.

PROBE_ID	SYMBOL	Probe_a11_14	Probe_11_aDetection Pval	aAld-aCort 1	Probe_b11_14	Probe_11_bDetection Pval	bAld-bCort 1	Probe_a12_14	Probe_12_aDetection Pval	Probe_b12_14	Probe_12_bDetection Pval
ILMN_2184373	**IL8**	57,229	0	**1820**	379,263	0	**1709**	-1762,565	0	-1329,587	0
ILMN_1666733	**IL8**	134,953	0	**1530**	891,414	0	**2222**	-1395,035	0	-1330,257	0
ILMN_2105573	**CCL3L3**	743,041	0	**942**	321,055	0	**785**	-198,847	0	-464,293	0
ILMN_2396020	**DUSP6**	422,315	0	**679**	103,579	0	**344**	-257	0	-240,6788	0
ILMN_2054297	**PTGS2**	411,68	0	**600**	279,271	0	**567**	-187,922	0	-288,12	0
ILMN_1775501	**IL1B**	227,11	0	**585**	255,286	0	**598**	-358,301	0	-343,192	0
ILMN_1682717	**IER3**	252,463	0	**511**	223,162	0	**652**	-258,3981	0	-429,2967	0
ILMN_1743199	**EGR2**	300,2283	0	**449**	230,5368	0	**327**	-148,3334	0	-96,9516	0
ILMN_2150856	**SERPINB2**	179,3313	0	**252**	196,7889	0	**371**	-73,1149	0	-173,9853	0
ILMN_1671509	**CCL3**	89,9532	0	**242**	94,1784	0	**225**	-152,1989	0	-131,1847	0
ILMN_1693338	**CYP1B1**	91,3254	0	**178**	157,1789	0	**220**	-86,8942	0	-62,8305	0
ILMN_1711838	**SLC25A24**	192,7405	0	**173**	46,4786	0	**161**	19,5611	0	-114,9093	0
ILMN_1682636	**CXCL2**	134,857	0	**138**	146,8472	0	**190**	-3,5092	0	-43,0331	0
ILMN_1747355	**CCL3L1**	66,6357	0	**129**	111,3409	0	**209**	-62,7065	0	-97,4506	0
ILMN_1722781	**EGR3**	93,1818	0	**123**	78,1428	0	**102**	-29,444	0	-24,055	0
ILMN_2150851	**SERPINB2**	22,6272	0	**116**	78,4134	0	**168**	-93,7641	0	-89,9059	0
ILMN_1687978	**PHLDA1**	84,4136	0	**115**	125,4099	0	**164**	-30,608	0	-39,0718	0
ILMN_1723141	**OTUD1**	44,9881	0	**115**	48,1091	0	**113**	-69,8594	0	-64,9563	0
ILMN_2328666	**CD83**	56,774	0	**114**	25,014	0	**183**	-56,7494	0	-157,5498	0
ILMN_1806023	**JUN**	75,2724	0	**107**	56,8758	0	**112**	-32,2206	0	-54,9794	0
ILMN_1713178	**FAM116A**	47,4888	0	**106**	15,1976	0	**34**	-58,1337	0	-18,6843	0
ILMN_1728106	**TNF**	40,9612	0	**97**	26,7362	0	**100**	-56,1534	0	-73,2199	0
ILMN_1780582	**CD83**	150,111	0	**96**	171,207	0	**272**	54,39	0	-100,304	0
ILMN_1678833	**CCR1**	33,4375	0	**83**	4,3296	0	**106**	-50,0152	0	-101,7709	0
ILMN_2374865	**ATF3**	20,7254	0	**82**	48,9642	0	**77**	-61,7065	0	-28,2136	0
ILMN_1780546	**OSM**	22,523	0	**81**	42,3606	0	**112**	-58,0537	0	-69,8137	0
ILMN_2218856	**CCL3L1**	25,0336	0	**80**	96,394	0	**108**	-55,1115	0	-11,1373	0
ILMN_1778177	**ZNF207**	15,497	0	**68**	286,115	0	**253**	-52,184	0	32,889	0
ILMN_1815086	**NINJ1**	72,3274	0	**62**	3,3514	0	**59**	10,4649	0	-55,9853	0
ILMN_1774874	**IL1RN**	15,6482	0	**58**	16,9301	0	**48**	-42,1697	0	-31,5557	0

ILMN_1758811	**IMPA1**	13,0219	0	**52**	11,5904	0	**12**	-39,2141	0	-0,6383	0
ILMN_1710514	**BCL3**	40,4696	0	**52**	34,2802	0	**20**	-11,6331	0	14,144	0
ILMN_1757287	**MAPK6**	45,1659	0	**49**	79,8856	0	**60**	-3,6098	0	19,399	0
ILMN_1680223	**PNPLA8**	100,9895	0	**48**	106,9466	0	**49**	52,7059	0	58,1097	0
ILMN_1773245	**CCL3L1**	26,21242	0	**45**	85,51314	0	**95**	-18,56651	0	-9,74773	0
ILMN_2173291	**CYP4B1**	28,80739	0	**43**	17,47451	0	**16**	-14,47226	0	1,04913	0
ILMN_1712357	**HNRPK**	16,6441	0	**40**	11,3827	0	**27**	-23,64077	0	-15,6759	0
ILMN_1683969	**FKBP1A**	10,96	0	**39**	32,8796	0	**33**	-28,363	0	-0,5013	0
ILMN_1690352	**ADO**	45,9718	0	**39**	24,2135	0	**6**	6,8945	0	17,8462	0
ILMN_2183938	**LEMD3**	68,9963	0	**39**	41,6523	0	**29**	29,94	0	12,687	0
ILMN_1729691	**SLC16A6**	25,63753	0	**38**	31,30934	0	**29**	-12,1888	0	2,43345	0
ILMN_1813361	**ANGPTL7**	28,453344	0	**37**	27,06595	0,001298701	**33**	-8,3232167	0,5623376	-6,253914	0,7220779
ILMN_2069945	**SNRNP27**	78,6778	0	**36**	61,4503	0	**31**	42,8543	0	30,1045	0
ILMN_3242586	**RHOU**	22,0447	0	**36**	1,0317	0	**37**	-13,4891	0	-35,7505	0
ILMN_1658483	**IL1A**	25,78694	0	**35**	23,82127	0	**28**	-9,14383	0	-4,33271	0
ILMN_1720083	**EHD4**	14,4682	0	**31**	48,3092	0	**69**	-16,399	0	-20,7304	0
ILMN_1687785	**PPA2**	47,6833	0	**29**	52,1498	0	**23**	18,4238	0	29,2368	0
ILMN_1659415	**MAP2K1IP1**	28,6201	0	**23**	52,0236	0	**51**	5,2543	0	0,6203	0
ILMN_1793410	**SNTB1**	28,8083	0	**23**	4,2762	0	**18**	5,8763	0	-13,9656	0
ILMN_1782110	**ZNF295**	27,4864	0	**22**	11,6288	0	**8**	5,0917	0	3,4729	0
ILMN_1761456	**ALG13**	30,9453	0	**22**	48,005	0	**83**	8,6318	0	-35,2862	0
ILMN_1691111	**SPATA2L**	5,2214	0	**22**	18,0662	0	**31**	-16,343	0	-12,6819	0
ILMN_3237385	**NRBF2**	18,4654	0	**21**	16,8189	0	**10**	-2,1908	0	6,4904	0
ILMN_2203891	**SMAD7**	14,1607	0	**17**	28,4692	0	**33**	-3,192	0	-4,53962	0
ILMN_1800942	**KCTD6**	12,92006	0	**17**	4,44796	0	**7**	-4,23064	0	-2,40797	0
ILMN_1700413	**MAFF**	35,01915	0	**16**	53,30402	0	**51**	19,21492	0	2,17996	0
ILMN_1772743	**PIGK**	12,1381	0	**15**	1,5956	0	**3**	-3,2779	0	-1,8776	0
ILMN_1769388	**GJB2**	12,76079	0	**15**	21,19867	0	**14**	-1,92916	0	6,71503	0,001298701
ILMN_1766045	**SH3GLB1**	13,4764	0	**13**	0,5648	0	**46**	0,2581	0	-45,2045	0
ILMN_1720048	**CCL2**	1,70056	0	**13**	4,88172	0,002597403	**18**	-10,812209	0,1207792	-12,7455459	0,3909091
ILMN_1656118	**ZBTB7A**	0,40388	0	**12**	1,81959	0,001298701	**0**	-11,46288	0	1,53796	0,001298701
ILMN_1657248	**EREG**	10,61684	0	**12**	6,78888	0	**1**	-0,97808	0	6,03391	0
ILMN_1891857		19,93997	0	**11**	6,54603	0	**8**	9,12159	0	-1,16489	0
ILMN_1682935	**LYPLAL1**	12,21317	0	**11**	16,48049	0	**15**	1,67461	0	1,04629	0
ILMN_1786532	**CNIH**	0,1365	0	**10**	9,1618	0	**19**	-9,9418	0	-9,613	0
ILMN_1725169	**INTS12**	8,3704	0	**9**	21,652	0	**0**	-0,192	0	21,2851	0
ILMN_1723116	**AMFR**	5,732425	0,002597403	**9**	1,825024	0,01558442	**6**	-2,795394	0,08701298	-4,422573	0,1454545
ILMN_1797372	**C3orf58**	15,409	0	**8**	19,4433	0	**42**	7,3455	0	-22,1448	0
ILMN_1813314	**HIST1H2BK**	1,79071	0	**7**	0,72136	0	**7**	-4,88075	0	-6,4294	0
ILMN_1686937	**ETNK1**	5,2444407	0,06623377	**6**	3,4916139	0,1571429	**2**	-0,7886054	0,5272727	1,315994	0,3688312

ILMN_1655644	**ZNF804A**	5,366337	0,01558442	**6**	0,793399	0,04935065	**3**	-0,628076	0,2428571	-1,730043	0,1506494
ILMN_2048507	**KLF3**	6,14501	0	**6**	5,56217	0	**1**	0,35459	0	4,84056	0
ILMN_1704070	**BCL10**	0,80432	0,002597403	**6**	10,298386	0,005194805	**2**	-4,902035	0,05064935	7,969046	0,009090909
ILMN_1913478		1,4307552	0,2324675	**6**	1,59602777	0,4584416	**4**	-4,0976718	0,8402597	-2,146145	0,8441558
ILMN_1691403	**SYDE2**	1,683609	0,01168831	**5**	2,962424	0,02337662	**3**	-3,731355	0,1493506	-0,012305	0,08311688
ILMN_1783629	**C10orf93**	3,907647	0,2935065	**5**	4,009177	0,3090909	**2**	-1,467612	0,8727273	1,8454341	0,5571429
ILMN_1713266	**FAM46C**	114,554	0	**5**	61,4722	0	**87**	109,2824	0	-25,528	0
ILMN_1784256	**HDGFRP3**	0,61203	0	**5**	3,31171	0,001298701	**2**	-4,59303	0	1,21667	0,002597403
ILMN_1805410	**C15orf48**	12,41579	0	**5**	10,71893	0	**14**	7,26167	0	-3,58406	0,002597403
ILMN_1797130	**BAI1**	2,6420536	0,5194805	**4**	0,595026	0,7831169	**0**	-1,427266	0,9103896	0,320262	0,7883117
ILMN_3245756	**LUZP6**	8,582358	0,07532468	**4**	1,93111	0,04155844	**6**	4,576343	0,3480519	-3,8809727	0,3831169
ILMN_1785345	**GPR84**	6,44815	0	**4**	1,12901	0	**8**	2,45445	0	-6,7011	0
ILMN_1720322	**PTS**	6,69748	0	**4**	14,11787	0	**19**	3,04107	0	-5,02535	0
ILMN_1651752	**CXorf21**	1,38266	0,001298701	**4**	7,9957	0,002597403	**7**	-2,26037	0,009090909	1,25316	0,007792208
ILMN_1654141	**ZDHHC20**	14,116491	0	**3**	14,68487	0,001298701	**14**	11,099341	0	0,71581	0,01948052
ILMN_1856609		11,82741	0	**3**	0,4676	0	**2**	8,8288	0	-1,29049	0
ILMN_1724213	**PAGE2**	5,4151154	0,08311688	**3**	7,038698	0,2701299	**7**	2,5512854	0,2818182	0,27881	0,9220779
ILMN_1733847	**GALR2**	2,170334	0,0948052	**2**	3,9366417	0,1051948	**4**	-0,324798	0,2818182	0,3555216	0,3948052
ILMN_1794187	**FBXL3**	24,5759	0	**2**	34,4439	0	**33**	22,3608	0	1,5859	0
ILMN_2323848	**PARD6A**	2,379069	0,01428571	**2**	1,760993	0,01688312	**6**	0,280854	0,06103896	-4,365071	0,1675325
ILMN_1704485	**OR7A17**	2,172442	0,3545454	**2**	0,497482	0,2896104	**3**	0,136078	0,5922078	-2,934451	0,6623377
ILMN_1771447	**ZNF833**	3,3304121	0,2285714	**2**	6,649102	0,1415584	**3**	1,3968715	0,4142857	3,5706499	0,4350649
ILMN_1727975	**SNTG1**	5,239922	0,7	**2**	0,772933	0,8701299	**0**	3,57694	0,8844156	0,590889	0,8636364
ILMN_1716182	**GAB4**	3,728004	0,3415585	**2**	7,9433406	0,3922078	**1**	2,1930871	0,5012987	7,37248679	0,4779221
ILMN_1678191	**GDF10**	5,0524221	0,07922078	**2**	1,3716974	0,4155844	**1**	3,5445061	0,1714286	-0,0931872	0,6025974
ILMN_1739126	**TMEM213**	1,973735	0,6090909	**1**	1,6997925	0,2805195	**2**	0,477732	0,8012987	0,1752367	0,448052
ILMN_2170095	**PMCH**	4,792545	0,2753247	**1**	2,7300387	0,1961039	**7**	3,3063788	0,4168831	-4,5128593	0,9181818
ILMN_2155516	**QTRTD1**	19,8585	0	**1**	16,8451	0	**10**	18,399	0	7,1941	0
ILMN_1901305		3,206542	0,1285714	**1**	3,949057	0,04935065	**5**	1,789735	0,2363636	-0,871459	0,3428572
ILMN_1667018	**ACE2**	3,4273074	0,125974	**1**	0,102508	0,06363636	**3**	2,0672974	0,2298701	-2,605353	0,2220779
ILMN_2314169	**PTHLH**	1,545833	0,1350649	**1**	2,728801	0,3207792	**1**	0,554524	0,2246753	2,089763	0,3961039
ILMN_1807493	**ACVRL1**	1,964129	0,6961039	**1**	0,8023811	0,3246753	**4**	1,116589	0,8246753	-2,7307749	0,712987
ILMN_1710735	**ZNF451**	1,172837	0,6012987	**1**	2,9017807	0,3714286	**4**	0,425491	0,7142857	-1,478203	0,8337662
ILMN_2054261	**RFX6**	3,6930085	0,151948	**0**	3,310167	0,07012987	**6**	3,3945495	0,1831169	-2,209093	0,5324675
ILMN_2367233	**ZNF654**	3,79418	0	**0**	2,85407	0,001298701	**13**	3,65199	0	-9,8352	0,007792208

Table S3. Resulting gene list for proband 2 following the strategy as outlined in paragraph 3.1 and depicted in Figure S3. For understanding of sample IDs in column titles, refer to Figure S3. In short: ‚a‘ or ‚b‘ indicates MicroArray used (b being a technical replicate of a), the first digit designates the number of the proband and the second digit the incubation condition (1 = aldosterone, 2 = cortisol, 4 = vehicle). Normalisation to vehicle is indicated by adding the vehicles abbreviation respectively, e.g. Probe_a11_14. Further processing of data sets normalised to vehicle is designated per MicroArray and proband, e.g. aAld-aCort 1 meaning cortisol signals were subtracted from aldosterone signals. P detection values of <0.001 are rounded down to zero.

PROBE_ID	SYMBOL	Probe_a21_24	Probe_21_aDetection Pval	aAld-aCort 2	Probe_b21_24	Probe_21_bDetection Pval	bAld-bCort 2	Probe_a22_24	Probe_22_aDetection Pval	Probe_b22_24	Probe_22_bDetection Pval
ILMN_1775501	**IL1B**	808,572	0	**932**	553,818	0	**860**	-123,325	0	-305,879	0
ILMN_1780582	**CD83**	157,019	0	**322**	141,886	0	**252**	-164,82	0	-109,975	0
ILMN_2396020	**DUSP6**	55,218	0	**311**	125,813	0	**408**	-256,1079	0	-281,8371	0
ILMN_2328666	**CD83**	125,6854	0	**278**	58,1041	0	**238**	-152,4908	0	-180,3294	0
ILMN_2105573	**CCL3L3**	280,412	0	**225**	278,9455	0	**158**	55,783	0	121,0465	0
ILMN_2184373	**IL8**	2038,676	0	**202**	1599,739	0	**481**	1836,821	0	1118,426	0
ILMN_1693338	**CYP1B1**	120,989	0	**172**	149,9457	0	**124**	-50,5493	0	26,3795	0
ILMN_1682717	**IER3**	210,1378	0	**160**	80,7228	0	**140**	50,1492	0	-59,2034	0
ILMN_1713266	**FAM46C**	98,36	0	**152**	128,225	0	**197**	-53,83	0	-68,858	0
ILMN_1743199	**EGR2**	132,7203	0	**145**	218,6485	0	**202**	-12,1095	0	16,4523	0
ILMN_1778177	**ZNF207**	196,818	0	**138**	85,583	0	**91**	59,018	0	-5,571	0
ILMN_2054297	**PTGS2**	51,085	0	**117**	275,067	0	**276**	-66,348	0	-0,947	0
ILMN_1680223	**PNPLA8**	150,6975	0	**111**	124,724	0	**16**	39,735	0	108,3504	0
ILMN_1678833	**CCR1**	78,6309	0	**108**	86,9541	0	**105**	-29,3139	0	-18,2287	0
ILMN_2150856	**SERPINB2**	79,7139	0	**106**	79,0837	0	**122**	-26,5357	0	-43,1392	0
ILMN_1806023	**JUN**	102,9023	0	**102**	72,9305	0	**101**	1,3831	0	-28,4935	0
ILMN_1728106	**TNF**	45,6041	0	**101**	52,8129	0	**105**	-55,598	0	-51,88108	0
ILMN_1710514	**BCL3**	39,1187	0	**97**	14,7911	0	**86**	-57,3966	0	-71,6634	0
ILMN_2150851	**SERPINB2**	42,4491	0	**88**	19,7211	0	**75**	-45,88114	0	-55,76712	0
ILMN_1766045	**SH3GLB1**	23,2903	0	**88**	30,7834	0	**41**	-64,5209	0	-10,2632	0
ILMN_1780546	**OSM**	52,9604	0	**84**	22,012	0	**68**	-31,3281	0	-46,2626	0
ILMN_1774874	**IL1RN**	37,5	0	**83**	45,3559	0	**63**	-45,4052	0	-17,8604	0
ILMN_1747355	**CCL3L1**	59,5066	0	**76**	62,9944	0	**58**	-16,2289	0	4,9761	0
ILMN_1723141	**OTUD1**	92,6537	0	**75**	117,8153	0	**108**	17,7771	0	9,3427	0
ILMN_1815086	**NINJ1**	26,758	0	**73**	14,0961	0	**137**	-46,6868	0	-123,1838	0
ILMN_1723116	**AMFR**	81,8729	0	**71**	73,9442	0	**83**	10,5795	0	-9,1701	0
ILMN_2203891	**SMAD7**	7,7017	0	**64**	9,1072	0	**75**	-55,845	0	-66,1308	0
ILMN_3242586	**RHOU**	41,4083	0	**59**	28,2016	0	**80**	-17,8751	0	-52,1008	0
ILMN_1671509	**CCL3**	79,5954	0	**59**	57,7574	0	**61**	20,8644	0	-3,1774	0
ILMN_1687978	**PHLDA1**	62,8513	0	**54**	75,3641	0	**69**	8,9805	0	6,4276	0
ILMN_1711838	**SLC25A24**	86,7169	0	**52**	114,1258	0	**55**	34,9106	0	59,5501	0
ILMN_1761456	**ALG13**	78,9079	0	**47**	15,2524	0	**10**	31,55	0	4,8556	0
ILMN_1757287	**MAPK6**	96,0786	0	**42**	136,2445	0	**84**	53,6017	0	51,9968	0

ILMN_1794187	**FBXL3**	57,574	0	**39**	77,0888	0	**27**	18,0759	0	50,2613	0
ILMN_1722781	**EGR3**	49,2721	0	**39**	5,5519	0	**15**	10,7343	0	-9,6703	0
ILMN_2374865	**ATF3**	24,501	0	**38**	19,5576	0	**43**	-13,364	0	-22,9496	0
ILMN_1725169	**INTS12**	33,9368	0	**36**	94,8576	0	**100**	-2,1672	0	-5,2853	0
ILMN_1797372	**C3orf58**	33,4346	0	**36**	43,7627	0	**15**	-2,3681	0	29,178	0
ILMN_2218856	**CCL3L1**	65,86944	0	**35**	58,3828	0	**53**	30,37514	0	5,4771	0
ILMN_1658483	**IL1A**	27,35369	0	**34**	31,82063	0	**37**	-6,78886	0	-4,9051	0
ILMN_1786532	**CNIH**	27,677	0	**34**	31,4457	0	**35**	-6,09	0	-3,2902	0
ILMN_1713178	**FAM116A**	17,6023	0	**32**	72,299	0	**16**	-14,6524	0	56,0268	0
ILMN_1700413	**MAFF**	25,70287	0	**30**	15,70392	0	**9**	-4,51097	0	6,40464	0
ILMN_2173291	**CYP4B1**	31,01727	0	**30**	26,66702	0	**17**	1,11707	0	9,79217	0
ILMN_1691111	**SPATA2L**	11,7776	0	**30**	9,6736	0	**11**	-17,8547	0	-1,7917	0
ILMN_1720048	**CCL2**	13,45006	0	**29**	24,46113	0	**41**	-15,342306	0,03896104	-16,775085	0,3142857
ILMN_1720083	**EHD4**	14,2029	0	**27**	10,2276	0	**5**	-13,2652	0	4,7477	0
ILMN_2069945	**SNRNP27**	65,5255	0	**27**	116,6416	0	**67**	38,5227	0	49,3324	0
ILMN_1666733	**IL8**	727,383	0	**26**	1284,06	0	**223**	701,844	0	1061,197	0
ILMN_1805410	**C15orf48**	24,20883	0	**25**	13,89918	0	**23**	-0,70755	0	-9,41791	0,001298701
ILMN_1659415	**MAP2K1IP1**	79,1804	0	**24**	38,9598	0	**10**	55,0634	0	28,5556	0
ILMN_2183938	**LEMD3**	121,78	0	**24**	101,1025	0	**46**	97,8297	0	55,3524	0
ILMN_1683969	**FKBP1A**	21,1494	0	**21**	1,2927	0	**3**	0,6297	0	-1,6995	0
ILMN_1729691	**SLC16A6**	17,12935	0	**20**	10,49587	0	**16**	-2,45612	0	-5,90532	0
ILMN_1785345	**GPR84**	8,9278	0	**18**	6,16253	0	**18**	-8,91665	0,0012987	-11,77919	0,001298701
ILMN_1800942	**KCTD6**	15,28835	0	**18**	13,2831	0	**23**	-2,33368	0	-9,94514	0
ILMN_1712357	**HNRPK**	1,65792	0	**16**	1,5044	0	**17**	-14,42325	0	-15,89336	0
ILMN_1856609		1,60124	0	**16**	15,42412	0	**12**	-13,94304	0	3,35448	0
ILMN_1733847	**GALR2**	5,800742	0,002597403	**14**	2,890531	0,01298701	**5**	-8,5492018	0,4376623	-1,930575	0,06623377
ILMN_1793410	**SNTB1**	44,9919	0	**14**	44,5437	0	**28**	30,7073	0	16,8801	0
ILMN_1720322	**PTS**	4,05132	0	**13**	23,87848	0	**27**	-9,39034	0	-2,9545	0
ILMN_1758811	**IMPA1**	54,1835	0	**13**	26,4912	0	**16**	41,3645	0	10,7511	0
ILMN_3237385		**NRBF2**	16,4113	0	**13**	20,7715	0	**16**	3,7057	0	4,742
ILMN_1682636	**CXCL2**	87,2264	0	**12**	106,1891	0	**38**	75,1289	0	68,2344	0
ILMN_1813314	**HIST1H2BK**	3,92083	0	**12**	2,06098	0	**2**	-7,71878	0	0,2378	0
ILMN_1891857		2,03339	0	**11**	21,01017	0	**17**	-9,16504	0	4,09007	0
ILMN_2155516	**QTRTD1**	17,8903	0	**10**	29,84407	0	**13**	7,6527	0	16,64137	0
ILMN_1782110	**ZNF295**	16,5385	0	**10**	16,7586	0	**22**	6,3875	0	-5,3921	0
ILMN_1686937	**ETNK1**	3,474797	0,04935065	**9**	0,462371	0,1779221	**2**	-5,82755	0,7831169	-1,465917	0,3402597
ILMN_1772743	**PIGK**	12,0573	0	**8**	13,8093	0	**24**	3,7005	0	-10,6763	0
ILMN_1704070	**BCL10**	1,177091	0,009090909	**8**	9,990356	0,002597403	**7**	-6,969162	0,2987013	3,28005	0,01948052
ILMN_1784256	**HDGFRP3**	10,38544	0	**7**	11,77274	0	**0**	2,89213	0	11,35807	0
ILMN_2054261	**RFX6**	1,602489	0,05194805	**7**	6,73205004	0,04285714	**4**	-5,726515	0,5961039	3,16892204	0,161039

ILMN_1678191		**GDF10**	9,210196	0,04285714	**6**	5,20514	0,01428571	**8**	3,3468873	0,387013	-2,539543
ILMN_1769388	**GJB2**	11,78133	0	**6**	8,57089	0	**13**	6,02415	0	-4,17382	0,003896104
ILMN_1797130	**BAI1**	0,212225	0,08701298	**6**	2,354727	0,5038961	**2**	-5,3639862	0,512987	0,401345	0,7428572
ILMN_1913478		0,179962	0,3246753	**5**	2,3582672	0,3103896	**3**	-5,250872	0,8662338	-0,2083278	0,5974026
ILMN_2367233	**ZNF654**	4,67776	0,001298701	**5**	4,75072	0	**3**	-0,61441	0,0012987	1,58122	0,001298701
ILMN_1656118	**ZBTB7A**	4,70457	0	**5**	5,63609	0	**6**	-0,5147	0	-0,41042	0,002597403
ILMN_1813361	**ANGPTL7**	5,226902	0,04155844	**5**	2,8499774	0,1532468	**4**	0,077704	0,287013	-1,2593969	0,5506493
ILMN_1654141	**ZDHHC20**	12,660087	0,002597403	**5**	6,601301	0,002597403	**3**	7,515777	0,0025974	3,835801	0,01038961
ILMN_1773245	**CCL3L1**	7,05386	0	**5**	9,72743	0	**9**	2,3102	0	0,77689	0
ILMN_1691403	**SYDE2**	1,282012	0,005194805	**5**	4,755276	0,06103896	**7**	-3,440414	0,04805195	-2,744426	0,648052
ILMN_1687785	**PPA2**	27,5685	0	**5**	54,7638	0	**3**	22,8747	0	51,9071	0
ILMN_1651752	**CXorf21**	5,44278	0,002597403	**5**	5,02041	0,001298701	**2**	0,78405	0,0012987	2,9786	0,007792208
ILMN_1807493		**ACVRL1**	3,13173	0,2935065	**5**	2,011583	0,6909091	**4**	-1,466117	0,774026	-2,383267
ILMN_1783629	**C10orf93**	7,032884	0,09740259	**4**	0,518548	0,4896104	**5**	2,584275	0,4233766	-4,1301537	0,9311689
ILMN_2048507	**KLF3**	17,49674	0	**4**	16,23658	0	**5**	13,15133	0	11,57003	0
ILMN_1727975	**SNTG1**	4,717556	0,6090909	**4**	2,115392	0,7324675	**0**	0,590741	0,9428571	1,811323	0,7961039
ILMN_1724213	**PAGE2**	0,689914	0,8324676	**4**	3,8005732	0,4181818	**6**	-3,357811	0,9896104	-2,297661	0,9545454
ILMN_2170095	**PMCH**	5,890999	0,1883117	**4**	3,717514	0,7194805	**2**	2,0590776	0,5467532	1,813162	0,8961039
ILMN_1710735	**ZNF451**	4,496277	0,2662338	**3**	4,5340962	0,4818182	**1**	1,4829662	0,574026	3,488255	0,6051948
ILMN_1716182	**GAB4**	5,85464	0,1090909	**3**	2,5400617	0,1779221	**4**	3,086314	0,2831169	-1,8388433	0,6155844
ILMN_1657248	**EREG**	1,3342	0	**3**	6,20021	0	**5**	-1,41898	0	1,10809	0
ILMN_3245756	**LUZP6**	0,5813751	0,3207792	**3**	4,915125	0,01428571	**4**	-2,1513609	0,6064935	0,567819	0,06623377
ILMN_2323848	**PARD6A**	0,558528	0,01168831	**3**	0,598299	0,01428571	**7**	-2,112908	0,04545455	-6,564391	0,2012987
ILMN_1901305		5,9438163	0,09350649	**3**	1,5076498	0,3545454	**0**	3,4364103	0,2155844	1,3189163	0,3675325
ILMN_1682935	**LYPLAL1**	2,01353	0	**2**	15,11906	0	**2**	-0,44504	0	13,52026	0
ILMN_1690352		**ADO**	73,1153	0	**2**	91,1537	0	**21**	70,7923	0	70,2998
ILMN_1771447	**ZNF833**	9,021112	0,2272727	**1**	4,338436	0,1961039	**1**	7,553585	0,3480519	3,675562	0,2493507
ILMN_1655644	**ZNF804A**	0,18721	0,1948052	**1**	1,715441	0,1	**3**	-0,94408	0,2779221	-1,063648	0,2584416
ILMN_2314169	**PTHLH**	3,1945015	0,238961	**1**	1,2130528	0,5	**3**	2,4077685	0,2974026	-1,885042	0,8311688
ILMN_1667018	**ACE2**	4,1826013	0,1038961	**0**	5,657835	0,2337662	**4**	3,7168643	0,1194805	1,650541	0,6597403
ILMN_1704485	**OR7A17**	4,125598	0,2818182	**0**	1,9223176	0,225974	**9**	3,709744	0,3246753	-7,0519114	0,9805195
ILMN_1739126	**TMEM213**	1,013022	0,6025974	**0**	0,887825	0,6324675	**1**	0,635991	0,648052	-0,092549	0,7545455

Table S4. Resulting gene list for proband 3 following the strategy as outlined in paragraph 3.1 and depicted Figure S3. For understanding of sample IDs in column titles see Figure S3. In short: ‚a' or ‚b' indicates MicroArray used (b being a technical replicate of a), the first digit designates the number of the proband and the second digit the incubation condition (1 = aldosterone, 2 = cortisol, 4 = vehicle). Normalisation to vehicle is indicated by adding the vehicles abbreviation respectively, e.g. Probe_a11_14. Further processing of data sets normalised to vehicle is designated per MicroArray and proband, e.g. aAld-aCort 1 meaning cortisol signals were subtracted from aldosterone signals. P detection values of <0.001 are rounded down to zero.

PROBE_ID	SYMBOL	Probe_a31_34	Probe_31_aDetection Pval	aAld-aCort 3	Probe_b31_34	Probe_31_bDetection Pval	bAld-bCort 3	Probe_a32_34	Probe_32_aDetection Pval	Probe_b32_34	Probe_32_bDetection Pval
ILMN_1666733	**IL8**	1213,151	0	**900**	1126,106	0	**33**	313,493	0	1093,075	0
ILMN_2184373	**IL8**	1072,885	0	**706**	1619,824	0	**514**	366,969	0	1105,797	0
ILMN_2054297	**PTGS2**	192,985	0	**501**	7,894	0	**342**	-308,3907	0	-333,73	0
ILMN_2396020	**DUSP6**	139,9474	0	**481**	16,3951	0	**373**	-341,0685	0	-356,4383	0
ILMN_1775501	**IL1B**	572,543	0	**439**	610,246	0	**442**	133,141	0	168,523	0
ILMN_1743199	**EGR2**	263,8146	0	**283**	174,3078	0	**205**	-18,9277	0	-31,0359	0
ILMN_1780582	**CD83**	308,652	0	**250**	276,501	0	**195**	58,411	0	81,942	0
ILMN_1682717	**IER3**	321,2844	0	**229**	332,5275	0	**125**	92,2179	0	207,2012	0
ILMN_2105573	**CCL3L3**	534,496	0	**193**	422,368	0	**250**	341,715	0	172,146	0
ILMN_2328666	**CD83**	224,6963	0	**172**	123,7001	0	**107**	52,3388	0	16,8913	0
ILMN_1713266	**FAM46C**	122,2197	0	**143**	63,8803	0	**124**	-20,5842	0	-60,039	0
ILMN_1693338	**CYP1B1**	8,015	0	**138**	34,3308	0	**188**	-130,0262	0	-153,9265	0
ILMN_1728106	**TNF**	45,2555	0	**128**	60,6906	0	**137**	-82,5874	0	-75,89902	0
ILMN_1711838	**SLC25A24**	174,5568	0	**112**	81,444	0	**85**	62,2653	0	-3,5976	0
ILMN_1747355	**CCL3L1**	126,7865	0	**111**	192,1103	0	**122**	16,2163	0	70,5063	0
ILMN_2150856	**SERPINB2**	59,9136	0	**105**	54,3458	0	**80**	-45,5419	0	-25,9641	0
ILMN_1713178	**FAM116A**	15,4754	0	**100**	4,2456	0	**19**	-84,5557	0	-14,3613	0
ILMN_1671509	**CCL3**	157,0136	0	**91**	121,048	0	**105**	66,5015	0	15,7188	0
ILMN_3242586	**RHOU**	3,5902	0	**83**	63,8053	0	**68**	-79,4566	0	-4,6201	0
ILMN_1778177	**ZNF207**	172,907	0	**78**	160,803	0	**199**	94,54	0	-38,455	0
ILMN_1780546	**OSM**	37,0023	0	**77**	49,003	0	**94**	-40,10617	0	-45,22141	0
ILMN_1774874	**IL1RN**	48,543	0	**77**	73,247	0	**86**	-28,5007	0	-12,6238	0
ILMN_1687978	**PHLDA1**	85,5306	0	**71**	94,031	0	**66**	14,6036	0	27,9367	0
ILMN_1757287	**MAPK6**	35,1848	0	**68**	34,273	0	**37**	-33,0862	0	-3,1438	0
ILMN_1690352	**ADO**	42,7872	0	**67**	16,4436	0	**63**	-24,14	0	-46,2609	0
ILMN_2218856	**CCL3L1**	98,394	0	**63**	131,4714	0	**70**	35,1876	0	61,17	0
ILMN_1682636	**CXCL2**	124,2143	0	**54**	106,2594	0	**52**	70,4334	0	54,4627	0
ILMN_1678833	**CCR1**	29,1454	0	**53**	58,1189	0	**90**	-23,9621	0	-32,3497	0
ILMN_2150851	**SERPINB2**	27,78665	0	**47**	41,5319	0	**62**	-19,64035	0	-20,11304	0
ILMN_1806023	**JUN**	10,0455	0	**45**	36,4931	0	**65**	-34,7133	0	-28,8531	0
ILMN_1793410	**SNTB1**	49,5533	0	**45**	8,2345	0	**40**	4,8293	0	-31,634	0
ILMN_1723141	**OTUD1**	43,0664	0	**42**	41,4667	0	**96**	1,1625	0	-54,8632	0
ILMN_2069945	**SNRNP27**	41,9836	0	**41**	3,4497	0	**2**	1,3487	0	1,2176	0

ILMN_1710514	**BCL3**	20,9471	0	**39**	46,3984	0	**65**	-18,2989	0	-18,9908	0
ILMN_1761456	**ALG13**	31,8903	0	**38**	32,1224	0	**32**	-6,1508	0	0,4817	0
ILMN_1813361	**ANGPTL7**	34,550998	0	**36**	19,768967	0,001298701	**22**	-1,106291	0,3337662	-2,532599	0,287013
ILMN_1815086	**NINJ1**	27,0571	0	**34**	103,6201	0	**16**	-7,4223	0	87,9487	0
ILMN_1659415	**MAP2K1IP1**	6,7676	0	**34**	48,8435	0	**43**	-27,6106	0	5,9262	0
ILMN_2203891	**SMAD7**	17,65775	0	**34**	19,47811	0	**38**	-15,9816	0	-18,38056	0
ILMN_2155516	**QTRTD1**	1,9403	0	**30**	21,7884	0	**41**	-28,14612	0	-19,54033	0
ILMN_1700413	**MAFF**	22,50601	0	**30**	15,36763	0	**8**	-7,22304	0	7,28023	0
ILMN_1712357	**HNRPK**	44,16798	0	**26**	47,00372	0	**25**	18,41115	0	22,40192	0
ILMN_2173291	**CYP4B1**	25,46472	0	**25**	16,00296	0	**4**	0,7935	0	12,35031	0
ILMN_1797372	**C3orf58**	9,3938	0	**25**	21,0459	0	**52**	-15,2419	0	-31,3941	0
ILMN_2374865	**ATF3**	24,0931	0	**23**	44,8438	0	**54**	0,7645	0	-9,6275	0
ILMN_1680223	**PNPLA8**	7,8856	0	**22**	9,3427	0	**30**	-13,7493	0	-21,1529	0
ILMN_1786532	**CNIH**	17,9581	0	**21**	15,8077	0	**16**	-2,795	0	-0,2903	0
ILMN_1794187	**FBXL3**	25,64616	0	**21**	2,2556	0	**19**	4,89956	0	-17,2222	0
ILMN_2048507	**KLF3**	15,63574	0	**19**	10,38881	0	**14**	-3,59302	0	-4,07874	0
ILMN_1773245	**CCL3L1**	54,96453	0	**19**	40,10583	0	**13**	35,80566	0	27,05213	0
ILMN_1658483	**IL1A**	19,56144	0	**18**	20,02814	0	**25**	1,97713	0	-4,50441	0,006493506
ILMN_1720048	**CCL2**	5,21541	0	**17**	17,15761	0,001298701	**28**	-11,718597	0,1025974	-10,726492	0,3441558
ILMN_1723116	**AMFR**	26,652	0	**17**	1,4672	0	**4**	9,8323	0	-2,6777	0
ILMN_1800942	**KCTD6**	24,34629	0	**16**	6,36396	0	**5**	8,00954	0	0,88667	0
ILMN_1813314	**HIST1H2BK**	18,31142	0	**16**	27,21102	0	**9**	2,08771	0	17,94462	0
ILMN_1725169	**INTS12**	26,9528	0	**16**	21,8978	0	**12**	11,0024	0	9,626	0
ILMN_1785345	**GPR84**	14,85925	0	**16**	15,89067	0	**21**	-1,00242	0	-5,5729	0,003896104
ILMN_1720322	**PTS**	21,81771	0	**15**	13,43457	0	**15**	6,93445	0	-1,26902	0
ILMN_1729691	**SLC16A6**	26,81096	0	**15**	18,90633	0	**20**	12,2084	0	-1,27669	0
ILMN_1682935	**LYPLAL1**	5,23214	0	**14**	3,96194	0	**14**	-9,04796	0	-9,55621	0
ILMN_1687785	**PPA2**	4,2816	0	**14**	27,3774	0	**17**	-9,5097	0	10,3981	0
ILMN_1782110	**ZNF295**	14,46944	0	**13**	28,38932	0	**30**	1,58077	0	-1,1572	0
ILMN_1766045		**SH3GLB1**	3,0361	0	**11**	19,886	0	**6**	-8,1399	0	13,497
ILMN_1683969	**FKBP1A**	30,0827	0	**11**	66,8625	0	**22**	19,007	0	44,8614	0
ILMN_1722781	**EGR3**	64,31955	0	**11**	59,23905	0	**31**	53,32675	0	28,05155	0
ILMN_3237385	**NRBF2**	19,02725	0	**11**	14,64404	0	**9**	8,11031	0	5,45224	0
ILMN_1784256	**HDGFRP3**	3,16107	0	**10**	1,17414	0,001298701	**6**	-6,72732	0	-4,73896	0
ILMN_1667018	**ACE2**	7,0565524	0,02337662	**9**	3,80466736	0,1480519	**3**	-1,9825626	0,6623377	1,00268076	0,3805195
ILMN_1913478		6,2561541	0,05064935	**9**	4,3297888	0,3727273	**4**	-2,7747059	0,7818182	0,044633	0,812987
ILMN_1651752	**CXorf21**	2,58213	0	**8**	10,7125	0,001298701	**9**	-5,70388	0,001298701	2,15164	0,002597403
ILMN_1724213	**PAGE2**	8,845972	0,2012987	**8**	11,020378	0,1480519	**11**	0,996049	0,9272727	-0,073269	0,987013
ILMN_1856609		7,477	0	**8**	1,27501	0	**1**	-0,12978	0	0	0
ILMN_1656118	**ZBTB7A**	1,47629	0	**7**	9,1758	0	**6**	-5,96387	0	2,84614	0

ILMN_1733847	**GALR2**	2,200562	0,01298701	**7**	0,12671	0,1311688	**2**	-5,170574	0,3402597	-1,78139
ILMN_1704485	**OR7A17**	4,1902945	0,1064935	**7**	1,225329	0,2077922	**1**	-3,1763855	0,7753247	0,565399	0,2753247
ILMN_1891857		3,53756	0	**7**	10,24143	0	**10**	-3,66641	0	0,22133	0
ILMN_1807493	**ACVRL1**	2,807235	0,638961	**7**	1,724091	0,8571429	**3**	-4,221395	0,9948052	-1,148854	0,9831169
ILMN_1704070	**BCL10**	2,61689	0,0025974	**6**	10,564908	0,005194805	**13**	-3,863192	0,02597403	-2,551816	0,07402597
ILMN_1654141	**ZDHHC20**	7,821118	0,0025974	**6**	0,173438	0,02467532	**2**	1,341358	0,01038961	-2,154918	0,04155844
ILMN_1686937	**ETNK1**	7,539005	0,1207792	**6**	2,1726419	0,2363636	**1**	1,936963	0,6077922	1,0650939	0,338961
ILMN_2314169	**PTHLH**	6,3312856	0,07012987	**5**	2,9990179	0,1857143	**1**	0,8614458	0,4688312	2,4409879	0,2350649
ILMN_2183938	**LEMD3**	11,714	0	**5**	34,7742	0	**19**	6,5878	0	15,4356	0
ILMN_2054261	**RFX6**	2,874714	0,03896104	**5**	12,523461	0,006493506	**11**	-2,244177	0,3103896	2,016874	0,07662338
ILMN_2367233	**ZNF654**	6,33542	0	**5**	4,56848	0,002597403	**5**	1,62333	0	0,06146	0,005194805
ILMN_1710735	**ZNF451**	2,298068	0,1636364	**5**	2,858359	0,3519481	**3**	-2,357223	0,5987013	-0,43813	0,7064935
ILMN_1805410	**C15orf48**	9,32853	0	**4**	3,64214	0,001298701	**18**	4,91439	0	-14,151913
ILMN_1727975	**SNTG1**	1,684811	0,7311688	**4**	0,545685	0,7142857	**5**	-2,590094	0,974026	-4,930267	0,9883117
ILMN_1901305		6,313936	0,1025974	**4**	3,0525787	0,2220779	**3**	2,5191456	0,3805195	0,0343844	0,5220779
ILMN_1772743	**PIGK**	15,6913	0	**4**	1,3035	0	**12**	11,9604	0	-10,3328	0
ILMN_1771447	**ZNF833**	3,335253148	0,1805195	**4**	2,4535593	0,2688312	**3**	-0,385165552	0,5194805	-0,3804302	0,5571429
ILMN_1691111	**SPATA2L**	5,4272	0	**3**	22,795	0	**17**	1,9726	0	6,0484	0
ILMN_1657248	**EREG**	6,79279	0	**3**	1,13809	0,002597403	**3**	3,40731	0	-1,88521	0,002597403
ILMN_1678191	**GDF10**	1,1402865	0,4246753	**3**	2,8101335	0,1623377	**4**	-2,1961658	0,7948052	-1,5184523	0,5532467
ILMN_1716182	**GAB4**	4,441605	0,2350649	**3**	5,7609541	0,09090909	**5**	1,3082247	0,5662338	0,4634866	0,4974026
ILMN_1783629	**C10orf93**	2,98250492	0,1948052	**3**	2,1872344	0,3519481	**8**	0,05093892	0,4636364	-5,7424076	0,9818182
ILMN_2323848	**PARD6A**	2,554182	0,01298701	**3**	10,0836754	0,02207792	**3**	-0,003494	0,05454545	6,8122334	0,04025974
ILMN_1691403	**SYDE2**	2,849008	0,01558442	**2**	3,052592	0,08961039	**4**	0,513859	0,06103896	-1,1154284	0,3766234
ILMN_1758811	**IMPA1**	6,2909	0	**2**	15,0853	0	**7**	4,0452	0	7,8445	0
ILMN_1797130	**BAI1**	3,161473	0,3181818	**2**	4,28436721	0,4805195	**5**	1,1067051	0,5493507	-0,446058
ILMN_3245756	**LUZP6**	1,020184	0,2181818	**2**	0,267478	0,3194805	**1**	-1,0286901	0,4246753	-0,7969942	0,4415585
ILMN_2170095	**PMCH**	5,571807	0,274026	**2**	5,125962	0,3519481	**2**	3,6104952	0,4779221	3,4675233	0,5272727
ILMN_1769388	**GJB2**	2,18575	0	**2**	8,91253	0,001298701	**11**	0,58317	0	-1,94369	0,002597403
ILMN_1739126	**TMEM213**	0,373962	0,7155844	**1**	2,075588	0,3571429	**2**	-0,865381	0,812987	-0,139845	0,5974026
ILMN_1655644	**ZNF804A**	3,061302	0,04285714	**1**	3,155587	0,09350649	**5**	1,921725	0,07662338	-1,5309909	0,4428571
ILMN_1720083	**EHD4**	3,3544	0	**0**	22,2047	0	**22**	3,2346	0	0,7008	0

Table S5. List of putative MR marker genes that were consistently upregulated by stimulation with aldosterone in all three probands.

105_Diff>40	Proband 1	Proband 2	Proband 3	Synonyms
	**CCL3**	**CCL3**	**CCL3**	SCYA3; MIP-1-alpha; G0S19-1; LD78ALPHA; MIP1A
	**CCL3L1**	**CCL3L1**	**CCL3L1**	SCYA3L1; LD78BETA; 464.2; SCYA3L; D17S1718; LD78; G0S19-2; MGC104178; MGC12815
	**CCL3L3**	**CCL3L3**	**CCL3L3**	SCYA3L1; LD78BETA; 464.2; SCYA3L; D17S1718; LD78; MGC12815
	**CCR1**	**CCR1**	**CCR1**	HM145; MIP1aR; CKR-1; CD191; CMKBR1; SCYAR1
	**CD83**	**CD83**	**CD83**	HB15; BL11
	**CD83**	**CD83**	**CD83**	HB15; BL11
	**CYP1B1**	**CYP1B1**	**CYP1B1**	GLC3A; CP1B
	**DUSP6**	**DUSP6**	**DUSP6**	MKP3; PYST1
	**EGR2**	**EGR2**	**EGR2**	KROX20; FLJ14547; DKFZp686J1957; CMT4E; CMT1D
	**IER3**	**IER3**	**IER3**	IEX-1L; PRG1; IEX1; GLY96; DIF-2; IEX-1; DIF2
	**IL1B**	**IL1B**	**IL1B**	IL1F2; IL1-BETA; IL-1
	**IL1RN**	**IL1RN**	**IL1RN**	ICIL-1RA; IRAP; IL-1ra3; MGC10430; IL1F3; IL1RA
	**IL8**	**IL8**	**IL8**	GCP-1; MDNCF; NAP1; CXCL8; AMCF-I; TSG-1; LYNAP; NAP-1; MONAP; GCP1; SCYB8; LUCT; K60; LECT; 3-10C; b-ENAP; NAF
	**JUN**	**JUN**	**JUN**	AP1; c-Jun
	**MAPK6**	**MAPK6**	**MAPK6**	HsT17250; PRKM6; p97MAPK; ERK3; DKFZp686F03189
	**OSM**	**OSM**	**OSM**	MGC20461
	**OTUD1**	**OTUD1**	**OTUD1**	
	**PHLDA1**	**PHLDA1**	**PHLDA1**	DT1P1B11; PHRIP; TDAG51; MGC131738
	**PTGS2**	**PTGS2**	**PTGS2**	PHS-2; COX-2; hCox-2; PGHS-2; PGG/HS; COX2
	**SERPINB2**	**SERPINB2**	**SERPINB2**	PAI; HsT1201; PLANH2; PAI-2; PAI2
	**SERPINB2**	**SERPINB2**	**SERPINB2**	PAI; HsT1201; PLANH2; PAI-2; PAI2
	**SLC25A24**	**SLC25A24**	**SLC25A24**	DKFZp586G0123; APC1; SCAMC-1
	**TNF**	**TNF**	**TNF**	TNFSF2; DIF; TNF-alpha; TNFA
	**ZNF207**	**ZNF207**	**ZNF207**	DKFZp761N202
**105_log>0.05**	Proband 1	Proband 2	Proband 3	
	**CCL2**	**CCL2**	**CCL2**	SMC-CF; MCP1; MCAF; HSMCR30; GDCF-2; SCYA2; HC11; MGC9434; MCP-1; GDCF-2 HC11
	CCL3	CCL3	CCL3	SCYA3; MIP-1-alpha; G0S19-1; LD78ALPHA; MIP1A
	CCL3L1	CCL3L1	CCL3L1	SCYA3L1; LD78BETA; 464.2; SCYA3L; D17S1718; LD78; G0S19-2; MGC104178; MGC12815
	CCL3L1	CCL3L1	CCL3L1	
	CCL3L1			
	CCR1	CCR1	CCR1	HM145; MIP1aR; CKR-1; CD191; CMKBR1; SCYAR1
	CYP1B1	CYP1B1	CYP1B1	GLC3A; CP1B
	**CYP4B1**	**CYP4B1**	**CYP4B1**	P-450HP
	DUSP6	DUSP6	DUSP6	MKP3; PYST1
	EGR2	EGR2	EGR2	KROX20; FLJ14547; DKFZp686J1957; CMT4E; CMT1D
	IER3	IER3	IER3	IEX-1L; PRG1; IEX1; GLY96; DIF-2; IEX-1; DIF2
	**IL1A**	**IL1A**	**IL1A**	IL1F1; IL1-ALPHA; IL1; IL-1A

	IL1B	IL1B	IL1B	IL1F2; IL1-BETA; IL-1
	IL1RN	IL1RN	IL1RN	ICIL-1RA; IRAP; IL-1ra3; MGC10430; IL1F3; IL1RA
	JUN	JUN	JUN	AP1; c-Jun
	OSM	OSM	OSM	MGC20461
	PHLDA1	PHLDA1	PHLDA1	DT1P1B11; PHRIP; TDAG51; MGC131738
	SERPINB2	SERPINB2	SERPINB2	PAI; HsT1201; PLANH2; PAI-2; PAI2
	SERPINB2	SERPINB2	SERPINB2	PAI; HsT1201; PLANH2; PAI-2; PAI2
	TNF	TNF	TNF	TNFSF2; DIF; TNF-alpha; TNFA
